# Comparative Therapeutic Effects of Natural Compounds Against *Saprolegnia* spp. (Oomycota) and *Amyloodinium ocellatum* (Dinophyceae)

**DOI:** 10.3389/fvets.2020.00083

**Published:** 2020-02-21

**Authors:** Perla Tedesco, Paola Beraldo, Michela Massimo, Maria Letizia Fioravanti, Donatella Volpatti, Ron Dirks, Roberta Galuppi

**Affiliations:** ^1^Department of Veterinary Medical Sciences, Alma Mater Studiorum-University of Bologna, Bologna, Italy; ^2^Department of Agricultural, Food, Environmental and Animal Sciences, University of Udine, Udine, Italy; ^3^Future Genomics Technologies BV, Leiden, Netherlands

**Keywords:** *Amyloodinium ocellatum*, *Saprolegnia* sp., *in vitro* test, natural compounds, alternative treatments

## Abstract

The fish parasites *Saprolegnia* spp. (Oomycota) and *Amyloodinium ocellatum* (Dinophyceae) cause important losses in freshwater and marine aquaculture industry, respectively. The possible adverse effects of compounds used to control these parasites in aquaculture resulted in increased interest on the search for natural products with antiparasitic activity. In this work, eighteen plant-derived compounds (2′,4′-Dihydroxychalcone; 7-Hydroxyflavone; Artemisinin; Camphor (1R); Diallyl sulfide; Esculetin; Eucalyptol; Garlicin 80%; Harmalol hydrochloride dihydrate; Palmatine chloride; Piperine; Plumbagin; Resveratrol; Rosmarinic acid; Sclareolide; Tomatine, Umbelliferone, and Usnic Acid) have been tested *in vitro*. Sixteen of these were used to determine their effects on the gill cell line G1B (ATCC®CRL-2536™) and on the motility of viable dinospores of *Amyloodinium ocellatum*, and thirteen were screened for inhibitory activity against *Saprolegnia* spp. The cytotoxicity results on G1B cells determined that only two compounds (2′,4′-Dihydroxychalcone and Tomatine) exhibited dose-dependent toxic effects. The highest surveyed concentrations (0.1 and 0.01 mM) reduced cell viability by 80%. Upon lowering the compound concentration the percentage of dead cells was lower than 20%. The same two compounds revealed to be potential antiparasitics by reducing in a dose-dependent manner the motility of *A. ocellatum* dinospores up to 100%. With respect to *Saprolegnia*, a Minimum Inhibitory Concentration was found for Tomatine (0.1 mM), Piperine and Plumbagin (0.25 mM), while 2′,4′-Dihydroxychalcone considerably slowed down mycelial growth for 24 h at a concentration of 0.1 mM. Therefore, this research allowed to identify two compounds, Tomatine and 2′,4′-Dihydroxychalcone, effective against both parasites. These compounds could represent promising candidates for the treatment of amyloodiniosis and saprolegniosis in aquaculture. Nevertheless, further *in vitro* and *in vivo* tests are required in order to determine concentrations that are effective against the considered pathogens but at the same time safe for hosts, environment and consumers.

## Introduction

*Saprolegnia* spp. Nees, 1823 (Oomycota–Straminopila supergroup) and *Amyloodinium ocellatum* Brown, 1931 (Dinophyceae–Alveolata) are considered dangerous parasites in the management and the economy of freshwater and marine aquaculture industry, as they parasitize generally the skin and gills of fish (1, 2) and, concerning *Saprolegnia*, also the eggs (3), causing significant losses.

*Amyloodinium ocellatum* is an ectoparasite dinoflagellate of brackish and marine warm water fish worldwide. Amyloodiniosis can be a major threat for land-based and lagoon-type rearing sites causing a parasitic branchitis associated with high morbidity, mortality and significant economic losses (1, 4, 5).

Oomycetes of the genus *Saprolegnia*, in particular *S. parasitica*, are the causative agents of Saprolegniosis, a disease characterized by the presence of visible white or gray patches of filamentous mycelium on the skin of freshwater fish and their eggs, with high mortality rates in freshwater fish culture. Besides being a problem for the fish farming industry, *S. parasitica* has also been implicated in wild salmon population decline around the world (6, 7).

The control of these parasites is problematic and a variety of treatments has been investigated over time (8–13).

In the past, Saprolegniosis was controlled with Malachite Green (MG). Following the discovery of its potential carcinogenicity, teratogenicity and mutagenicity in humans, and its environmental impact, the use of MG in the production of fish destined to human consumption is not authorized in the European Union (EU) (14). Nevertheless, EFSA established that food contaminated with MG or its metabolic product leucomalachite green (LMG) at or below the reference point for action (RPA) of 2 μg/kg is unlikely to represent a public health concern (15).

Copper sulfate is the most widely used compound for the control of Amyloodiniosis epidemics, usually administered at 0.12–0.15 mg/L for 10–14 days, although its therapeutic doses are also toxic to most invertebrates and algae (1). Copper sulfate was also described as effective against *Saprolegnia*, both *in vitro* (16) and on fish eggs in flow-through systems (17). However, although not banned, this compound is not registered for treatment against *Saprolegnia* or *A. ocellatum* in European Union. High copper concentration in water is associated with acute toxicity in the gill, with loss of the structural integrity of the epithelium and of branchial ionoregulatory functions (18). Moreover, the use of copper sulfate in aquaculture can contribute to increased accumulation of copper in the soil, where it already tends to increase due to its use in agriculture, which poses a significant risk to human health (19).

Another commonly used compound for the treatment of both *Saprolegnia* and *A. ocellatum* is formalin, a 37% solution of formaldehyde. Fajer-Ávila et al. (20), surveying the antiparasitic properties of the compound against ectoparasites (among which *A. ocellatum*) of bullseye puffer fish (*Sphoeroides annulatus*), reported 4 mg/L as the dose at which the compound had less adverse effects on fish in long-term exposure and reduced the parasite burden on skin and gills after 7 h of treatment. With respect to *Saprolegnia*, formalin has been effectively employed for egg disinfection in aquaculture to both treat and prevent infection in fish eggs (21). These authors observed that it is capable of inhibiting *Saprolegnia* cyst germination at a concentration of 250 mg/L. Formalin is currently not licensed as a veterinary medicine for the treatment of live fish in most of the EU countries. From the 1st of January 2016, formaldehyde has been classified as a category 1B carcinogen (22), thus its use should be restricted, due to possible hazardous effects to both exposed workers and environment.

Therefore, the use of chemicals in aquaculture may generate problems of environmental contamination: particularly, effluents from aquaculture facilities have become the subject of major concern regarding pollution, with adverse effects on the treated organisms and the environment. For these reasons, research has been increasingly focused on the selection of natural products with antimicrobial activity, in order to reduce the impacts of chemical and synthetic compounds on the environment, and with the perspective to avoid the possible development of drug resistance in parasites.

The purpose of this comparative study was to investigate the *in vitro* effectiveness of different biological compounds, of proven antimicrobial, antifungal and antiparasitic activity, against the oomycete species *Saprolegnia parasitica* and *S. delica* and against *A. ocellatum* dinospores.

## Materials and Methods

### Origin of the Natural Compounds

According to the Consortium of the ParaFishControl Horizon2020 project, ZF-screens (ZF-S; Leiden, the Netherlands) provided a library from which 18 natural compounds were selected based on their recognized antiparasitic and antimicrobial activity. The compounds were supplied dissolved in Dimethyl Sulfoxide (DMSO) (10 mM) and stored at −20°C until use. Table 1 lists the natural compounds tested in the different experiments: 16 for cytotoxicity and activity against *A. ocellatum*, 13 for activity against *Saprolegnia* spp.

**Table 1:** List of natural compounds and different targets tested.

**Compounds**	**Concentration 0.1 mM (μg/ml)**	**G1B cell line**	***Amyloodinium ocellatum***	***Saprolegna* spp**.
2′,4′-Dihydroxychalcone^a^	24.03	+	+	+
7-Hydroxyflavone^b^	23.82	+	+	+
Artemisinin^b^	28.23	+	+	–
Camphor (1R)^b^	15.22	+	+	+
Diallyl sulfide^b^	11.42	+	+	+
Esculetin^b^	17.81	+	+	+
Eucalyptol^b^	15.43	+	+	+
Garlicin 80%^b^	16.25	+	+	–
Harmalol hydrochloride dihydrate^b^	27.27	+	+	–
Palmatine chloride^a^	38.79	+	+	+
Piperine^b^	28.53	+	+	+
Plumbagin^a^	18.82	–	–	+
Resveratrol^b^	22.82	+	+	–
Rosmarinic acid^b^	36.03	+	+	–
Sclareolide^b^	25.38	+	+	+
Tomatine^b^	99.41	+	+	+
Umbelliferone^b^	16.21	+	+	+
Usnic Acid^b^	86.08	–	–	+

a *Santa Cruz Biotechnology, Inc., Dallas, Texas USA*.

b *Sigma Aldrich, St. Louis, MO, USA*.

### Natural Compounds and Their Cytotoxicity in G1B Cell Line

Pre-trial tests were performed on the G1B gill cell line (ATCC^®^ CRL-2356™) in order to define the work compound concentrations and exclude the toxic ones. Cells were cultivated in sterile conditions in L-15 complete medium (L-15 containing 2 mM L-glutamine, 10% FCS, penicillin 100 U/ml, streptomycin 100 μg/ml) at 25°C. Upon reaching 100% confluence, cells were enzymatically digested with 0.25% Trypsin EDTA, counted by using the Trypan blue exclusion method and a Thoma chamber (23) and adjusted to a concentration of 1.1 × 10^6^/ml. The cells where then transferred into sterile 96-well tissue culture plates and incubated in L-15 complete medium in sterile conditions at 25°C for 24 h to let them adhere to the wells bottom. After this period, the supernatant was removed and replaced with the diluted plant compounds.

Sixteen compounds (Table 1) were tested three times in triplicate wells in sterile 96-well tissue culture plates (Sarstedt) at 10-fold serial dilutions (ranging from 0.1 to 0.000001 mM). Compounds were diluted in L-15 complete medium starting from a concentration at which the toxicity due to DMSO solvent was negligible. Then 225 μl of the diluted compounds were added to the wells with seeded cells and incubated for 24 h at 25°C. Control wells were always included and represented by cells incubated with only L-15 complete medium. After the incubation period, the cytotoxicity effects of the compounds were verified by the absorbance-based Neutral Red test as described by Taju et al. (24).

### *In vitro* Tests on *Amyloodinium ocellatum* Dinospores (AOd)

*In vitro* motility test was performed in two subsequent rounds: in the first round, AOd hatched from non-preserved tomonts were used and in the second round tomonts hibernated for 3 months before use, as described hereafter. Trophonts were collected from the gills of the same naturally infested European sea bass (*Dicentrarchus labrax*) following the protocol of Beraldo et al. (25). Then, purified early tomonts (before the first division) were aliquoted and part of them incubated at 24°C for immediate use, while some aliquots were maintained in a hibernation status (16°C) and used 3 months later, after transferring them to 24°C for AOd hatching (25, 26). The reproductive process of both non-hibernated and hibernated tomonts was constantly monitored under an inverted microscope to check for dinospore hatching capacity. No differences in the hatching rate of tomonts and in the morphology, vitality and motility of dinospores were seen.

Dinospores were counted by adapting Dehority's (27) protocol. Briefly, 500 μl aliquots of the dinospore suspensions were stained with Lugol's iodine solution and counted using a counting cell chamber (S50 Sedgewick Rafter Cell, Pyser–SGI). Parasites were adjusted to a concentration of 5,200 dinospores/ml in HBSS/IO2 medium [H8264, Sigma Aldrich (28)].

Sixteen natural compounds (Table 1) were tested twice in duplicate wells in sterile 96-well plates (Sarstedt), at 2-fold serial dilutions (ranging from 50 to 6.25 μg/ml; 100 μl/well). Then, the experiment was repeated only for the compounds with evident inhibitory properties; in this case the dilutions ranged from 50 to 0.39 μg/ml. The compounds were diluted in HBSS/IO2 medium starting from a concentration at which the toxicity due to DMSO solvent was negligible and from the cytotoxicity results on the G1B gill cell line.

Dinospores (100 μl) were then added to each well; those incubated in culture medium without compound were used as negative controls. Copper sulfate chelated with citric acid (100 μl/well) at a final concentration of 1 μg/ml and 100 μl/well of formalin (4 μg/ml) were included in some wells of the plates as inhibitory positive controls. Plates, covered with lids, were maintained at room temperature for the whole duration of the experiment (24 h).

For the motility tests, AOd behavior was observed under an inverted microscope. At 1, 6, and 24 h after the beginning of the incubation with the compounds, aliquots of 50 μl were taken from each well and transferred into urinary deposit chambers (Vacutest Kima precision cell) in order to facilitate the visualization and counting of the dinospores. Aliquots were inspected under light microscopy. Only non-motile dinospores and those whose flagella did not vibrate (dead dinospores) were counted. Based on the number of non-motile dinospores, the percentage of motile dinospores was calculated for every compound concentration and at the different times of evaluation. Motility percentages were then compared to the motility of dinospores detected in the negative control, to which a motility value of 100% was assigned.

### *In vitro* Tests on *Saprolegnia* spp.

Tests were carried out on three *Saprolegnia* strains: one reference strain of *Saprolegnia parasitica* (CBS 223.65 provided by CSIC-RJB Madrid, Spain) isolated in Holland from northern pike (*Esox lucius*), one field strain of *S. parasitica* (ITT 320/15/20) isolated in Italy from brown trout (*Salmo trutta fario*), and one field strain of *Saprolegnia delica* (ITT 290/15/15) isolated in Italy from rainbow trout (*Oncorhynchus mykiss*). *Saprolegnia* spp. strains were maintained with periodic subcultures on glucose-yeast (GY) agar medium (5 g D-(+)-glucose, 1 g yeast extract, 12 g agar in 1 L deionized water) supplemented with 6 mg/L of penicillin and 10 mg/L of oxolinic acid (GY + P + Ox) (29) and kept at 18°C. For the *in vitro* trials, subcultures of the strains employed were incubated at 18°C until growth covered the full diameter of the dish (48–72 h). Inocula were obtained from the outer 10 mm of the culture, using a sterile 5 mm diameter glass cannula (protocol I) or cutting a 4 × 4 mm piece with a sterile scalpel.

Thirteen compounds were tested (Table 1) following protocol I according to Alderman (30). Ten-fold serial dilutions of the compound were added to sterile GY agar maintained liquid at a temperature of 49°C to obtain final concentrations of 0.00001; 0.0001; 0.001; 0.01; 0.1; and 0.25 mM. Mixtures were then distributed in six-well plates (Ø 35 mm) (allowing to test 5 different concentrations and one negative control) and were let solidify overnight. A 5 mm diameter well was then obtained in the center of the agar, using a sterile glass cannula, and was filled with a standard 5 mm inoculum, culture surface facing upwards. Each strain was tested in triplicate per each concentration. 2′,4′-Dihydroxychalcone, 7-Hydroxyflavone, Eucalyptol, Esculetin, Palmatine Chloride and Tomatine were not tested at the concentration of 0.25 mM, since they were already slightly toxic at 0.1 mM, according to the results of cytotoxicity tests on G1B cells. Pure DMSO was also screened at the same amount to exclude any inhibitory or lethal effect. Plates were incubated at 18°C and checked after 24, 48, 72 h and 6 days, determining the colony diameter of the growing mycelium as average of two axes measured at 90° from each other. Mycelial growth was then expressed as mean value of the replicates. The minimum inhibitory concentration (MIC) was defined as the lowest concentration inhibiting completely the growth of the mycelium after 6 days of incubation.

## Results

### Cytotoxicity Test on G1B Cells

Cytotoxicity results demonstrated that 14 out of 16 compounds (7-Hydroxyflavone; Artemisinin; Camphor (1R); Diallyl sulfide; Esculetin; Eucalyptol; Garlicin 80%; Harmalol hydrochloride dihydrate; Palmatine chloride; Piperine; Resveratrol; Rosmarinic acid; Sclareolide and Umbelliferone) displayed no or slightly toxic effects on G1B cells (with <20% of dead cells). On the other hand, 2′,4′-Dihydroxychalcone and Tomatine exhibited dose-dependent toxic effects. At the highest concentrations investigated (0.1 and 0.01 mM) only 20% of the cells was still viable. However, the percentage of dead cells considerably decreased at the third concentration (0.001 mM) with more than 80% of viable cells, while the lowest concentrations displayed a negligible toxicity.

### *In vitro* Tests on Viable Dinospores of *Amyloodinium ocellatum*

**Figure 1:**
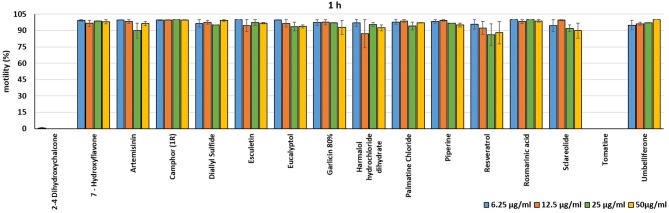
Motility of dinospores of *A. ocellatum* incubated at room temperature with different concentrations (6.25–50 μg/ml) of 16 plant derived compounds. The motility is expressed as percentage of motile dinospores on the total number of dinospores present per well after 1 h of incubation. Then, values have been related to the motility observed in the wells assigned to negative control and corresponding to 100%.

**Figure 2:**
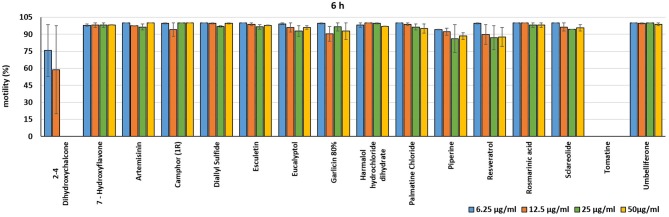
Motility of dinospores of *A. ocellatum* incubated at room temperature with different concentrations (6.25–50 μg/ml) of 16 plant derived compounds. The motility is expressed as percentage of motile dinospores on the total number of dinospores present per well after 6 h of incubation. Then, values have been related to the motility observed in the wells assigned to negative control and corresponding to 100%.

**Figure 3:**
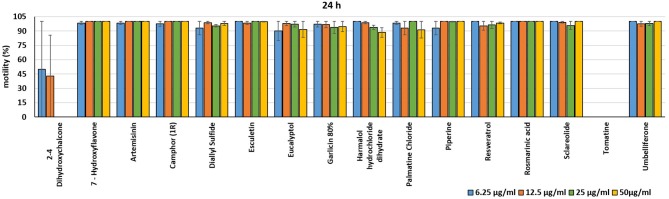
Motility of dinospores of *A. ocellatum* incubated at room temperature with different concentrations (6.25–50 μg/ml) of 16 plant derived compounds. The motility is expressed as percentage of motile dinospores on the total number of dinospores present per well after 24 h of incubation. Then, values have been related to the motility observed in the wells assigned to negative control and corresponding to 100%.

In the present investigation, the 16 plant compounds were initially tested twice on dinospore motility at concentrations ranging from 50 to 6.25 μg/ml. For these tests it was decided to express the concentration as μg/ml in order to study their properties at the same concentration of active compound. The range of tested concentrations derived from literature consultation and was applied in parallel studies to evaluate the possible immunomodulatory activities of the same compounds on European sea bass head kidney leukocytes by respiratory burst tests (31). Fourteen out of 16 compounds (7-Hydroxyflavone; Artemisinin; Camphor (1R); Diallyl sulfide; Esculetin; Eucalyptol; Garlicin 80%; Harmalol hydrochloride dihydrate; Palmatine chloride; Piperine; resveratrol; Rosmarinic acid; Sclareolide; and Umbelliferone) showed no considerable effects on dinospore motility, as described in Figures 1–3. In fact, the percentage of dinospores' movements in the wells with these substances was comparable to those recorded for the negative control.

**Figure 4:**
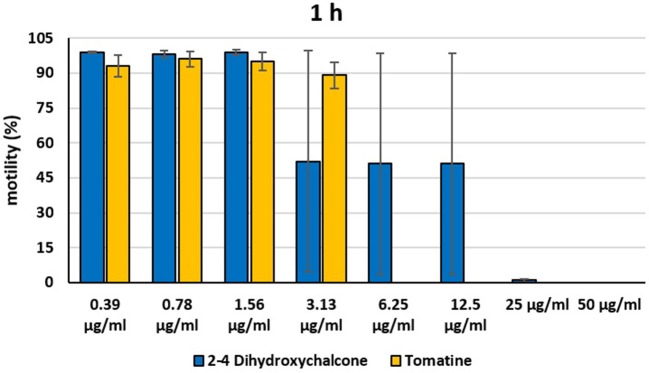
Motility of dinospores of *A. ocellatum* incubated at room temperature with different concentrations (0.39–50 μg/ml) of 2′,4′-dihydroxychalcone and tomatine. The motility is expressed as percentage of motile dinospores on the total number of dinospores present per well after 1 h of incubation. Then, values have been related to the motility observed in the wells assigned to negative control and corresponding to 100%.

**Figure 5:**
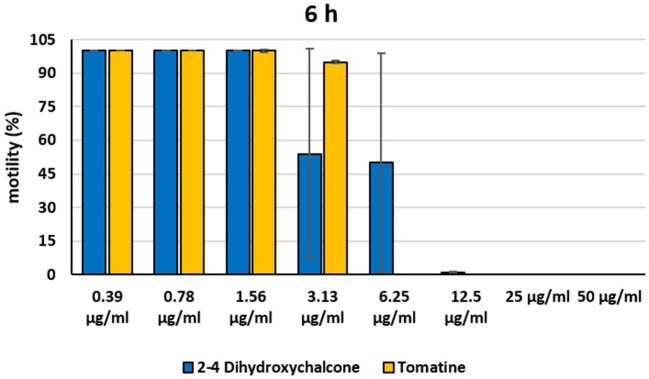
Motility of dinospores of *A. ocellatum* incubated at room temperature with different concentrations (0.39–50 μg/ml) of 2′,4′-dihydroxychalcone and tomatine. The motility is expressed as percentage of motile dinospores on the total number of dinospores present per well after 6 h of incubation. Then, values have been related to the motility observed in the wells assigned to negative control and corresponding to 100%.

**Figure 6:**
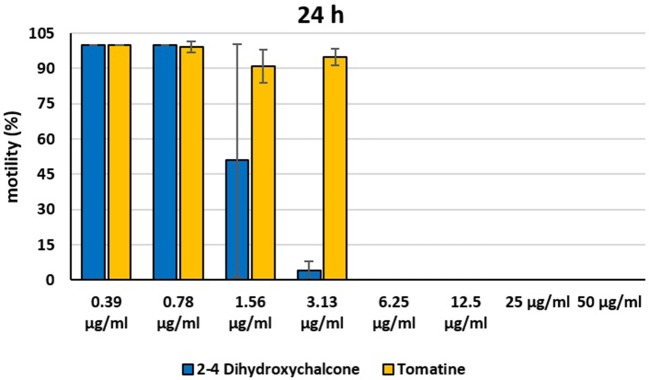
Motility of dinospores of *A. ocellatum* incubated at room temperature with different concentrations (0.39–50 μg/ml) of 2′-4′-dihydroxychalcone and tomatine. The motility is expressed as percentage of motile dinospores on the total number of dinospores present per well after 24 h of incubation. Then, values have been related to the motility observed in the wells assigned to negative control and corresponding to 100%.

Instead, 2′,4′-Dihydroxychalcone and Tomatine had an evident inhibitory action on dinospores, for this reason they were subsequently tested extending the range of concentrations from 50 to 0.39 μg/ml, as reported in Figures 4–6. These results showed that 2′,4′-Dihydroxychalcone inhibited the dinospore motility at the highest tested concentrations 50 and 25 μg/ml (0.2 and 0.1 mM) after 1 h of incubation. After 6 h of incubation, only the three highest doses 50, 25, and 12.5 μg/ml (0.2, 0.1, and 0.05 mM) stopped the motility of dinospores, whereas motility inhibition was halved at 6.25 and 3.13 μg/ml (0.025 and 0.013 mM), while the remaining concentrations did not demonstrate a relevant inhibitory activity on dinospore motility. After 24 h of incubation, 2′,4′-Dihydroxychalcone at the concentration range of 50–6.25 μg/ml (0.2–0.025 mM) inhibited the motility of all the dinospores in the wells, while at 3.13 μg/ml (0.025 mM) 4% of dinospores still moved. Conversely, 0.39, 0.78, and 1.56 μg/ml (0.002, 0.003, 0.006 mM) concentrations of this chalcone did not inhibit dinospore motility.

Tomatine displayed more evident inhibitory effects on dinospore motility than 2′,4′-Dihydroxychalcone for the whole duration of the experiment (24 h) in the range doses 6.25–50 μg/ml (0.006–0.05 mM). In fact, after 1 h of incubation, the highest concentrations of this substance ranging from 50 to 6.25 μg/ml (0.05–0.006 mM) completely inhibited the dinospore motility. On the other hand, in the concentrations ranging from 3.13 to 0.39 μg/ml (0.003–0.0004 mM) the percentage of swimming dinospores was 89–95%. Similarly, after 6 h and 24 h of incubation no motility of dinospores was observed in the wells with the highest Tomatine concentrations 50–6.25 μg/ml (0.05–0.006 mM) as noticed after 1 h, whereas the lowest concentrations were less or not effective, and capable of inhibiting dinospore motility up to 9%.

Chelated copper sulfate at 1 μg/ml (0.006 mM) was effective for the whole duration (24 h) of the experiment; similarly, formalin at 4 μg/ml (0.133 mM) concentration showed an inhibiting activity on the dinoflagellate but at a long term exposure. In fact, after 1 h of incubation only 2% of dinospores was immotile. However, at 6 and 24 h no swimming dinospores were observed.

### *In vitro* Tests on *Saprolegnia* spp.

According to the results of *in vitro* tests against *Saprolegnia* strains, triplicates were always consistent among each other. As expected, DMSO alone had no detectable effect at tested concentrations.

**Figure 7:**
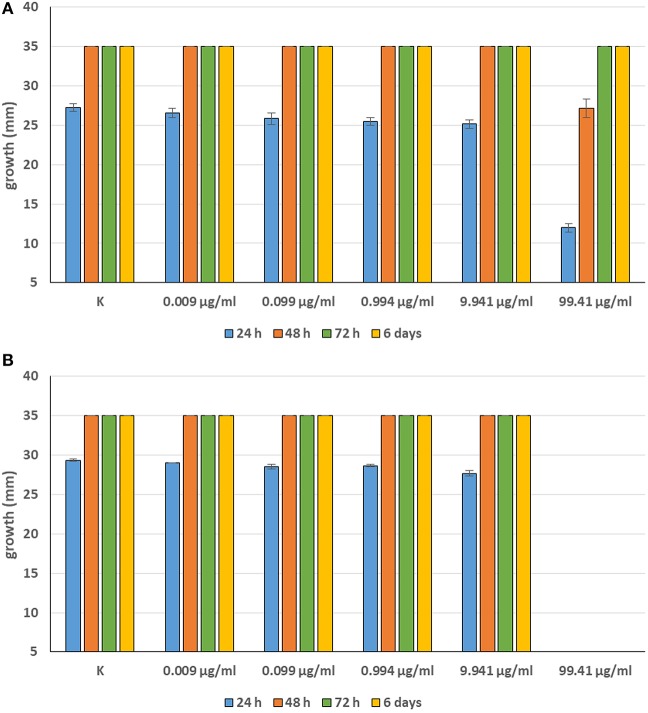
Average diameter (mm) of *Saprolegnia parasitica* **(A)** and *Saprolegnia delica* **(B)** mycelium with different concentrations of Tomatine.

**Figure 8:**
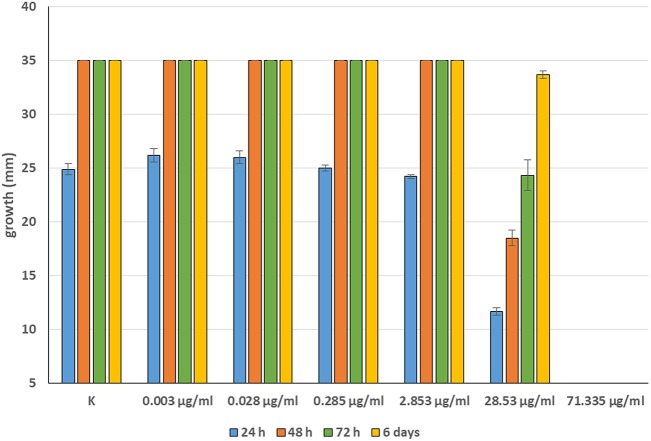
Average diameter (mm) of all tested strains mycelium with different concentrations of Piperine.

**Figure 9:**
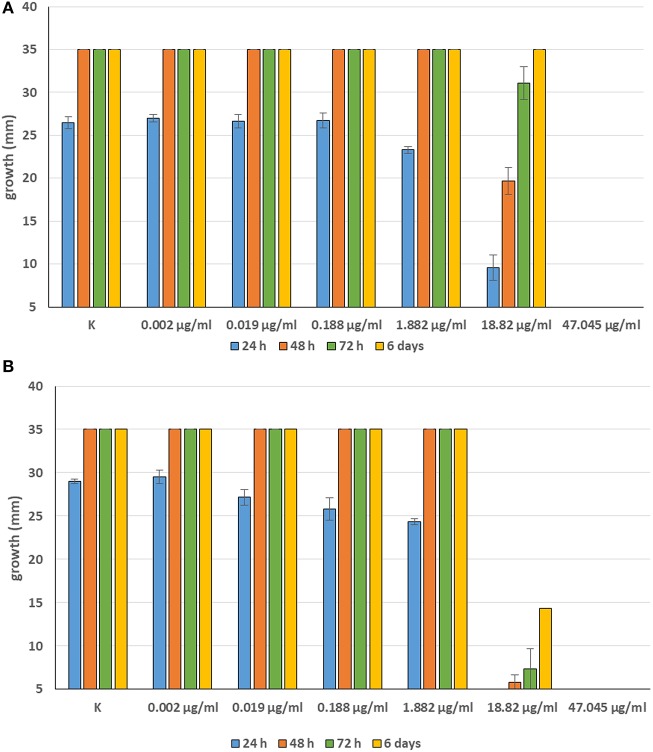
Average diameter (mm) of *Saprolegnia parasitica* **(A)** and *Saprolegnia delica* **(B)** mycelium with different concentrations of Plumbagin.

Minimum Inhibitory Concentrations (MICs) were defined for only three biological compounds, namely Tomatine, Piperine, and Plumbagin (Table 2), albeit with different *in vitro* behavior. Tomatine at 0.1 mM (99.4 μg/ml) completely inhibited *S. delica* while, for *S. parasitica*, at this concentration only a partial reduction of radial mycelial growth was observed (Figure 7). Nevertheless, after 6 days at this concentration, inhibition of aerial mycelium for *S. parasitica* was observed. The effect of Piperine, showing MIC at 0.25 mM (71.33 μg/ml), was consistent between different strains (Figure 8). Also Plumbagin showed MIC at 0.25 mM (47.045 μg/ml), although a complete inhibition of *S. delica* was also observed at a concentration of 0.1 mM (18.82 μg/ml) for the first 24 h (Figure 9).

**Table 2:** Results of the *in vitro* test of the 14 natural compounds against *Saprolegnia* spp.

**Compounds**	**MIC mM (μg/ml)**	**Note about protocol I**
2′,4′-Dihydroxychalcone	>0.1	After 6 days radial mycelial growth was considerably slowed down at 0.1 mM (24.020 μg/ml**)**
7-Hydroxyflavone	>0.1	
Camphor (1R)	>0.25	After 6 days inhibition of aerial mycelium at 0.25 mM (38.06 μg/ml**)**
Diallyl sulfide	>0.25	After 6 days inhibition of aerial mycelium at 0.25 mM (28.55 μg/ml**)**
Esculetin	>0.1	
Eucalyptol	>0.1	
Palmatine chloride	>0.1	
Piperine	0.25 (71.335)	
Plumbagin	0.25 (47.045)	
Sclareolide	>0.25	After 6 days radial mycelial growth was considerably slowed down at 0.25 mM (62.595 μg/ml**)** for *S. parasitica*; inhibition of aerial mycelium at 0.1 mM (25.038 μg/ml**)** for *S. parasitica* and 0.25 for *S. delica*
Tomatine	0.1 (99.4) (only for *S. delica)*	After 6 days inhibition of aerial mycelium at 0.1 mM (99.4 μg/ml) for *S. parasitica*
Umbelliferone	>0.25	After 6 days inhibition of aerial mycelium at 0.1 mM (16.214 μg/ml) for *S. parasitica* and at 0.25 mM (40.535 μg/ml) for *S. delica*
Usnic acid	>0.25	After 6 days radial mycelial growth was considerably slowed down at 0.25 mM (86.08 μg/ml)

**Figure 10:**
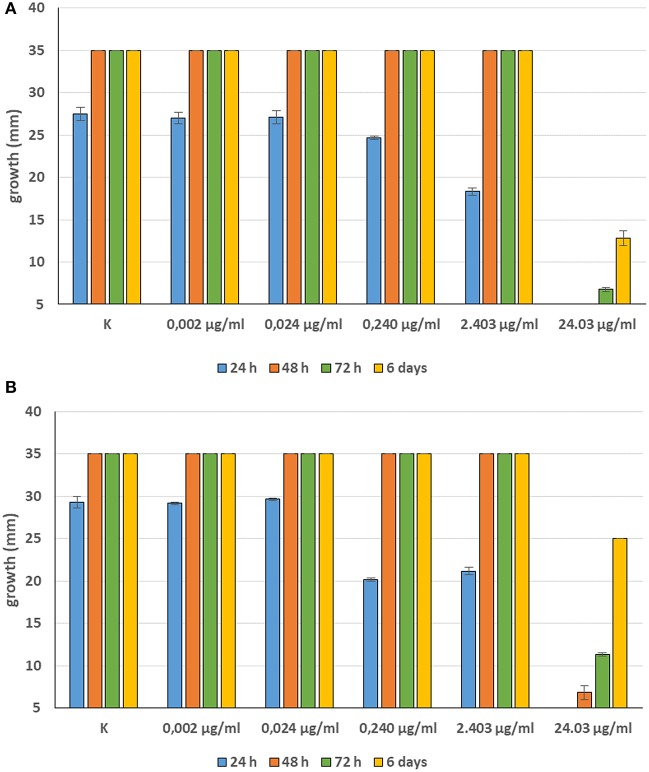
Average diameter (mm) of *Saprolegnia parasitica* **(A)** and *Saprolegnia delica* **(B)** mycelium with different concentrations of 2′,4′-Dihydroxychalcone.

For 2′,4′-Dihydroxychalcone no MIC was defined, however mycelial growth was considerably slowed down for 24 h in all tested strains at a concentration 0.01 mM (2.40 μg/ml). Furthermore, the complete inhibition of *S. parasitica* for 48 h and of *S. delica* for 24 h at a concentration of 0.1 mM (24.03 μg/ml) was observed; after 6 days, at this concentration mycelial growth was still slower than the control in all tested strains (Figure 10).

**Figure 11:**
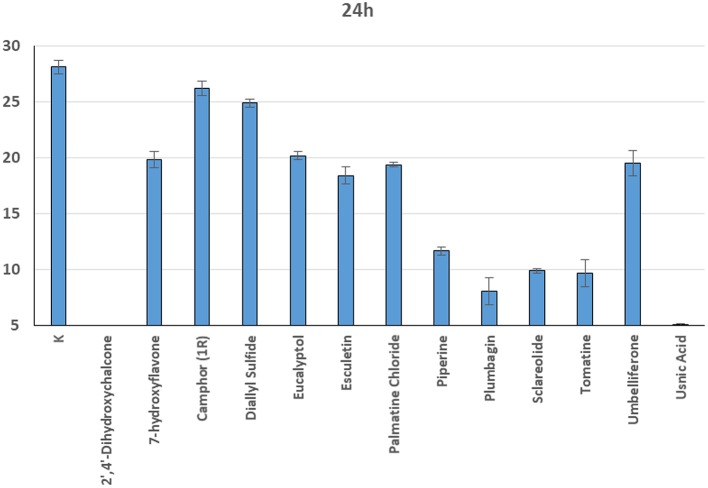
Average radial growth (mm) at 24 h of *Saprolegnia* spp. with different products at a concentration of 0.1 mM.

In general, a slower radial mycelial growth of *S. parasitica* and *S. delica* strains was recorded at 24 h for all tested compounds at a concentration 0.1 mM, although with different degrees of effectiveness (Figure 11). Percentage radial growth compared with the untreated control is reported in Table 3. Increasing concentrations of 7-Hydroxyflavone, Eucalyptol and Esculetin progressively slowed down mycelial growth only for 24 h, however at 48 h mycelial growth was equal to the control for all tested concentrations. A similar pattern occurred with Camphor and Diallyl Sulfide: increasing concentrations of these compounds progressively slowed down mycelial growth for 24 h at all tested concentrations, and for 48 h at a concentration of 0.25 mM (38.06 and 28.56 μg/ml, respectively). For Piperine and Sclareolide, mycelial growth of *S. parasitica* never reached the diameter of the control at 0.1 mM (28.53 and 25.38 μg/ml, respectively). The same growth pattern could be observed for Umbelliferone at a concentration of 0.25 mM (40.53 μg/ml). On the contrary, plumbagin was more effective at inhibiting the growth of *S. delica* at 0.1 mM (18.82 μg/ml) rather than the two tested *S. parasitica* strains. Moreover, Usnic Acid effectively reduced mycelial growth for 72 h at a concentration of 0.1 mM (86.08 μg/ml). Finally, at 6 days, in five compounds other than Tomatine, the inhibition of aerial mycelium was also observed at different concentrations: Camphor and Diallyl Sulfide at 0.25 mM (30.06 and 20.55 μg/ml, respectively) for all tested strains; Umbelliferone at 0.1 mM (16.21 μg/ml) for *S. parasitica* and at 0.25 mM (40.53 μg/ml) for *S. delica*; Sclareolide at 0.1 mM (25.038 μg/ml) for S. parasitica and 0.25 (62.59) for *S. delica* (Table 2).

**Table 3:** Percentage radial growth of *S. delica* e *S. parasitica* compared with the control after 24 h at 0.1 mM.

**Compounds**	**% Growth *S. parasitica***	**% Growth *S. delica***
2′,4′-Dihydroxychalcone	0	0
7-hydroxyflavone	67	69
Camphor (1R)	83	79
Diallyl sulfide	79	83
Eucalyptol	74	83
Esculetin	72	60
Palmatine chloride	69	78
Piperine	35	31
Plumbagin	21	0
Sclareolide	24	29
Tomatine	31	0
Umbelliferone	67	73
Usnic acid	0	0

## Discussion

The use of chemicals is commonly the principal approach for keeping ectoparasites in cultured fish under control. However, as highlighted above, compounds commonly used for the control of parasitic diseases in aquaculture are potentially harmful to fish, humans and the environment. In this context, there is a strong need to search for new strategies to limit the spread of diseases in fish farms, with a particular attention on animal welfare and environmental impact including, among other approaches, the use of natural compounds.

Regarding *Saprolegnia*, several studies are present in literature about the use of natural compounds. Tampieri et al. (32) evaluated the *in vitro* effectiveness of 18 essential oils and their isolated chemical constituents against *Saprolegnia parasitica*, finding that the most effective essential oils showed inhibitory and lethal effects at concentrations of 100 ppm (μg/ml). Moreover, plant extracts have been tested *in vitro* for their antifungal activity against *Saprolegnia ferax* isolated from naturally infected carps (*Cyprinus carpio*) (33), highlighting how a range of extracts displayed strong antifungal activity, at concentrations <500 μg/ml.

Regarding *Amyloodinium*, several treatments alternative to copper sulfate have been tested in experimental trials in order to manage amyloodiniosis, but inhibitory effects were not determined and, in some cases, neither fully clarified (9, 11). On the other hand, to date there is no available literature information about the *in vitro* and/or *in vivo* use of plant derived compounds or medicinal plants as antiparasitic candidates against *A. ocellatum*.

In the present study, the potential antiparasitic effects of 18 plant derived compounds were investigated. In particular, 13 compounds were screened to test their effect on the hyphal growth of *S. parasitica* and *S. delica*, while 16 compounds were tested on viable *A. ocellatum* dinospores (Table 1). In fact, among different developmental stages of *A. ocellatum*, only dinospores are susceptible to fish-safe chemical bath treatments (9, 13, 34), while trophonts and tomonts (the parasitic and reproductive stage of the protozoan, respectively) are more resistant. In the tests performed in the present investigation, copper sulfate and formalin were used as inhibitory positive control for *A. ocellatum*. Copper sulfate was used chelated with citric acid since the free copper ion, that is the active component, is unstable in seawater. Therefore, in order to increase the ion stability the usage of chelated copper compounds is recommended (1, 35). According to Paperna (9) and Bessat and Fadel (13), at a concentration of 1 μg/ml the compound was capable of completely interrupting dinospore motility for the whole duration of the experiment (24 h). Formalin was used at a concentration of 4 μg/ml on the basis of Fajer-Ávila et al. (20) *in vivo* investigations.

The choice of the compounds, selected from a larger pre-existing panel, was determined by their antiparasitic/anti-invertebrate activity in other contexts. For example, several studies have demonstrated antifungal activity of camphor-rich essential oils against a wide range of phytopathogenic fungi including the oomycetes *Phytophthora infestans* through alterations in hyphal morphology and cytoplasmic content (36). Garlic (*Allium sativum*) and its extracts have long been known for their antibacterial, antiviral, antifungal, antiparasitic, and immune modulating properties (37–43) and Buchmann et al. (44) found that garlic extract killed theronts of the ciliate *Ichthyophthirius multifiliis*. The antimicrobial activity of coumarins, among which Esculetin and Umbelliferone, has been demonstrated in several studies (45–50). Palmatine chloride has been recently screened against the causative agent of malaria, the protozoan parasite *Plasmodium falciparum*, showing strong anti-malarial effects (51). Flavonoids, such as 7-Hydroxyflavone, are naturally produced in plants with antifungal properties (52–54). Usnic acid, a secondary lichen metabolite, possesses antimicrobial activity against a number of planktonic gram-positive bacteria and seems to be able to inhibit bacterial biofilm formation (55). Piperine is a bioactive alkaloid found in the skin and seeds of black pepper (*Piper nigrum*) fruits. Earlier work highlighted the antibacterial and antifungal effectiveness of piperine suggesting its use as natural antimicrobial agent [(56, 57)]. Plumbagin is a hydroxyl-naphthoquinone originally extracted from plants belonging to the Plumbaginaceae with renown antifungal (58) and antiparasitic (59) properties.

Nevertheless, the *A. ocellatum* motility test results obtained in the present study, showed that 14 compounds [7-Hydroxyflavone; Artemisinin; Camphor (1R); Diallyl sulfide; Esculetin; Eucalyptol; Garlicin 80%; Harmalol hydrochloride dihydrate; Palmatine chloride; Piperine; Resveratrol; Rosmarinic acid; Sclareolide and Umbelliferone] had a limited inhibitory activity against the protozoan (up to 14%), while only the glycoalkaloid Tomatine and the chalcone 2′,4′-Dihydroxychalcone considerably reduced dinospores motility. Regarding *Saprolegnia* spp., even if a slower radial mycelial growth of *S. parasitica* and *S. delica* strains was recorded for all the 13 tested compounds, only 2′,4′-Dihydroxychalcone and Usnic Acid completely inhibited radial growth of both *Saprolegnia* spp. after 24 h at 0.1 mM (Table 3). MICs were determined only for Tomatine, Piperine and Plumbagin. Although no MIC was defined for 2′,4′-Dihydroxychalcone, after 6 days the mycelial growth was still reduced in all tested strains at 0.1 mM (24 μg/ml).

Glycoalkaloids, which are commonly referred to as saponins, are known to possess antimicrobial and antifungal activities that act as plant defenses against pests, pathogens and invasion by neighboring plants (60). Tomato plants (*Solanum lycopersicum*) produce Tomatine, a tetrasaccharide linked to the 3-OH group of the aglycone tomatidine. Immature green tomatoes contain up to 500 mg of Tomatine/kg of fresh fruit weight, nevertheless the compound is largely degraded as the tomato ripens until it reaches levels in mature red tomatoes of 5 mg/kg of fresh fruit weight (61). Tomatine administrated *in vivo* at a dose of 2,000 ppm showed anticarcinogenic effects against dibenzo[a,l]pyrene (DBP)-induced liver and stomach tumors in rainbow trout (*O. mykiss*) without adverse effects on animals (62). More recently, Liu et al. (63) documented inhibitory effects of Tomatine on human and animal pathogenic protozoa. In particular, the reported doses of the compound, which inhibited the growth of the three surveyed trichomonads, are in line with the concentrations tested in the present trial against *A. ocellatum*, in which Tomatine displayed inhibitory effects for the whole duration of the experiment (24 h) in the dose ranges 6.25–50 μg/ml (0.006–0.05 mM). Conversely, at concentrations lower than 6.25 μg/ml (0.006 mM) this glycoalkaloid was not or less effective. However, further studies will be necessary to understand whether Tomatine displays only an inhibitory effect or a dinosporicide activity against *A. ocellatum* at the surveyed effective concentrations. Previous research showed that α-tomatine owes its toxic properties to the ability to interact with 3ß-hydroxy sterol (64). Some oomycetes, such as *Pythium* and *Phytophthora* species, do not produce 3ß-hydroxy sterol and are relatively tolerant to α-tomatine (65). Nevertheless, Sandrock and VanEtten (66) found inhibitory effect on *Phytophthora infestans* and *Pythium aphanidermatum*, where this sterol is lacking, suggesting that α-tomatine may possess additional properties other than binding the 3ß-hydroxy sterol. In the present work, Tomatine showed a MIC of 99.4 μg/ml only against *Saprolegnia* delica although, after 6 days, at this concentration the inhibition of aerial mycelium for *S. parasitica* was observed. A similar behavior was also observed for Camphor (at 38 μg/ml), Diallylsulfide (at 28.6 μg/ml), Sclareolide (at 25 μg/ml for *S. parasitica* and 62.6 μg/ml for *S. delica*), and Umbelliferone (16.2 μg/ml for *S. parasitica* and 40.5 for *S. delica*) (Table 2). The inhibition of aereal mycelium of *Saprolegnia* spp. was also described, due to other compounds (67). In the past Kaminskyj and Heath (68) hypothesized that this phenomenon could be associated to chemically induced morphological hyphae changes, but the mechanisms of action are not clear and require further study.

The variety of biological properties displayed by chalcones is well-documented in literature. In fact, these aromatic ketones possess anti-inflammatory, antioxidant, antitumoral, antimicrobial, antifungal, anti-leishmanial, anti-malarial, and antiviral activities (69–71). 2′,4′-Dihydroxychalcone, isolated from *Zuccagnia punctata* Cav. (Fabaceae), has shown antifungal properties against a wide range of plant (72) and human (73) pathogens and has been suggested as promising antifungal agent for use in humans, acting by a different mechanism of action than currently used antifungal drugs, such as azoles or echinocandins (73). Seo et al. (74) tested the inhibitory effects and investigated the mode of action of 2′,4′-Dihydroxychalcone against *Aspergillus fumigatus*, demonstrating how the compound inhibits the calcinurin signaling pathway, necessary for proper hyphal growth. At 48 h, the MIC50 (MIC that inhibits 50% of growth) of 2′,4′-Dihydroxychalcone was between 64 and 128 μg/mL, while treatment with 256 μg/mL drastically decreased mycelial growth. This compound was tested also against *Saprolegnia* by Flores et al. (75), showing a 48 h MIC of 6.25 μg/mL. Partially in accord with these authors, our results show that 2′,4′-Dihydroxychalcone completely inhibited *S. parasitica* for 48 h and S. *delica* for 24 h at a concentration of 24.03 μg/mL (0.1 mM), however some mycelial growth was observed starting at 72 h. Conversely, the tests performed on *A. ocellatum* show that 2′,4′-Dihydroxychalcone completely inhibited the dinospore motility at the highest concentrations 50 and 25 μg/ml (0.2 and 0.1 mM) after 1 h of incubation, to then being inhibitory till 3.13 μg/ml (0.013 mM) in long term exposure (24 h). Anyway, other experiments are necessary to clarify if *A. ocellatum* dinospores may be killed by this compound at higher concentrations or if their motility is only temporarily inhibited in the presence of this substance. However, the results obtained by Flores et al. (75) with a spores germination inhibition test on *S. parasitica* and *S. australis*, suggest that 2′,4′-Dihydroxychalcone may display dinosporicide activity at concentrations higher than MIC, whereas at lower concentrations it could inhibit the dinospore movements only for a certain period of time. Nevertheless, since there are no published reports on the use of 2′,4′-Dihydroxychalcone in fish, further researches will be necessary to determine the effects of this compound on fish in the perspective of its application in aquaculture as remedy against fish parasites.

Only for *Saprolegnia* spp. an inhibiting activity was found also for Piperine and Plumbagin, at 71.3 μg/ml (0.25 mM) and 47 μg/ml (0.25 mM), respectively. In aquaculture, Piperine was also described as effective at 9.0 mg/L against *Argulus* sp. in *Carassius auratus* (76) however, to our knowledge, no studies involving *Saprolegnia* spp., are present in literature. The extract of *Plumbago rosea*, which contains plumbagin (2-methoxy-5-hydroxy-1-4-napthoquinone) as pharmacologically active component, was used mixed with food as immunostimulant in *Catla catla* post-challenged with *Aeromonas hydrophila* (77). Plumbagin was also found effective against various oomycetes (MIC = 5 μg/ml) but was not tested against *Saprolegnia* sp. (78). Other studies showed its considerable bactericidal activity and overall toxicity to aquatic organisms, and it was proposed for treatment of ship's ballast water (79). Therefore, its possible use in aquaculture should be subjected to further investigations aimed at excluding harmful effects on the environment.

To our knowledge, the plant derived compounds investigated in this study have never been used before on viable dinospores of *A. ocellatum* and only 2′,4′-Dihydroxychalcone was previously tested for *Saprolegnia* spp. (75). The present investigation showed that, among the compounds tested, Tomatine and 2′,4′-Dihydroxychalcone have shown to be effective for both parasites. In particular, even if the latter compound does not completely inhibit the mycelial growth of *Saprolegnia* spp. after 6 days, its possible application in aquaculture should be better investigated in the future. In fact, Flores et al. (75) stated that the use of 2′,4′-Dihydroxychalcone in the hatchery could be cost effective and safe for both workers and environment, representing a good option to replace synthetic compounds in the control of saprolegniosis. Nevertheless, the observations provided by this research are to be considered preliminary. Further *in vitro* investigations should be performed to better explore the mechanisms of action of these compounds, and *in vivo* small-scale trials should be arranged in order to select doses which may be effective against *A. ocellatum* and *Saprolegnia* spp. but at the same time non-toxic for their marine or freshwater hosts, respectively, the environment and consumer.

## Data Availability Statement

The datasets generated for this study are available on request to the corresponding author.

## Author Contributions

RG and PB conceived the original idea. PT, PB, MM, and DV carried out the *in vitro* tests. RD prepared and furnished the tested compounds solutions in DMSO. PT with RG, PB, and MM wrote the manuscript. MF supervised the project and all the authors provided critical feedback and the supervision of the manuscript.

### Conflict of Interest

RD was employed by company Future Genomics Technologies BV. The remaining authors declare that the research was conducted in the absence of any commercial or financial relationships that could be construed as a potential conflict of interest.
